# Does the island area also need to insist on salt iodization to prevent iodine deficiency disorders? a cross-sectional survey in Hainan Province, China

**DOI:** 10.3389/fendo.2025.1536506

**Published:** 2025-02-28

**Authors:** Hongying Wu, Shizhu Meng, Dingwei Sun, Yuting Hu, Tingou Wu, Xiaohuan Wang, Xingren Wang, Ying Liu, Chuyan Peng, Bin He, Fangang Meng

**Affiliations:** ^1^ Hainan Provincial Center for Disease Control and Prevention (Hainan Academy of Preventive Medicine), Haikou, Hainan, China; ^2^ Haikou Center for Disease Control and Prevention of Hainan Province, Haikou, Hainan, China; ^3^ Chengmai County Center for Disease Control and Prevention of Hainan Province, Chengmai, Hainan, China; ^4^ Center for Endemic Disease Control, Chinese Center for Disease Control and Prevention, Harbin Medical University, Harbin, Heilongjiang, China; ^5^ Key Lab of Etiology and Epidemiology, Education Bureau of Heilongjiang Province & Ministry of Health, Heilongjiang Provincial Key Lab of Trace Elements and Human Health, Harbin Medical University, Harbin, Heilongjiang, China

**Keywords:** island area, iodine status, salt iodization, iodine deficiency disorders, thyroid function

## Abstract

**Background:**

To investigate the epidemiology and related factors of iodine deficiency disorders (IDD) in Hainan Province, to know the iodine nutrition status and thyroid disease status of residents, and to explore whether salt iodization should be adopted to prevent and control IDD in island areas. To provide a basis for the effective implementation of scientific iodine supplement prevention and control strategy.

**Methods:**

All 21 cities, counties (districts) in the province were investigated. Superficial soil, residential drinking water, common food, urine samples of children, adults and pregnant women, household salt samples, thyroid B-ultrasound of adults and pregnant women, adult blood samples were collected. Soil iodine, water iodine, food iodine, urine iodine, daily salt intake, salt iodine, thyroid volume, nodules and thyroid function were measured.

**Results:**

The median iodine content in soil was 4.37mg/kg, the median iodine content in drinking water was 6.0μg/L, the iodized salt coverage rate was 98.6%, and the consumption rate of qualified iodized salt was 97.9%. The median urinary iodine concentration (MUIC) in children and adults was 180.3μg/L and 151.6μg/L, respectively, which was the adequate level of iodine. The median urinary iodine in pregnant women was 144.6μg/L, which was slightly lower than the adequate level. The main source of iodine intake was salt iodine, which contributed 59.8% to total dietary iodine. Kelp, milk and seaweed, whose contribution rates were 8.2%, 7.3% and 6.6%, respectively. The total iodine contribution rate of other foods was 18.1%, among which the contribution rate of fish, shrimp and crabs was only 2.4%. The overall prevalence of thyroid diseases was 27.01%. The detection rates of subclinical hypothyroidism and subclinical hyperthyroidism in males were significantly higher than those in females, and the detection rates of thyroid nodules and goiter were significantly lower than those in females. The detection rates of thyroid nodules in central mountainous areas were significantly higher than those in eastern and western coastal areas.

**Conclusions:**

At present, the iodine nutrition level in Hainan Province is generally in a suitable state, and the iodine intake of residents mainly comes from iodized salt. The strategy of salt iodization prevention and control of IDD should be adhered to in island area.

## 1 Introduction

Iodine deficiency disorders (IDD) is a general term for a group of diseases and hazards manifested by insufficient iodine intake, mainly including endemic goiter and endemic cretinism, overt hypothyroidism (1–3). Similarly, excessive iodine intake can also cause goiter, autoimmune thyroiditis, and increased detection rate of subclinical hypothyroidism (4, 5). Since 1995, China has implemented the policy of universal salt iodization for prevention and treatment of IDD, which has gradually solved the problem of iodine malnutrition and IDD in China for a long time. Since 2010, China has continued to eliminate IDD, providing experience for the prevention and treatment of IDD in other countries (6). Hainan Province is the southernmost provincial administrative region in China, surrounded by the sea, with a permanent population of 10.43 million. In recent years, there has been the spread of the opinion that “living by the sea is not short of iodine and excessive iodine supplement by salt causes increasing in thyroid diseases”. There are misunderstandings about the knowledge about iodine and thyroid diseases, and some residents have the mentality of “fearing salt iodization”, which has a certain impact on the prevention and treatment of IDD in China.

In order to understand the iodine intake level of residents in island province, the iodine nutrition status of the population, the relationship between iodine and thyroid disease, and whether to continue to eat iodized salt, we conducted a cross-sectional survey in all districts and counties of Hainan Province, providing scientific basis for the prevention and control of IDD in island areas, and providing new ideas for the prevention and control of IDD in coastal areas, so as to truly implement local conditions, classified guidance and scientific iodine supplementation.

## 2 Materials and methods

### 2.1 Survey areas

All the 21 cities and counties (districts) were selected in Hainan Province, and each survey county was divided into 5 sampling areas according to the east, west, south, north and middle, and 1 township/street is randomly selected in each area.

### 2.2 Survey content and sample collection

Currently, all Chinese people no longer adopt other specific measures to supplement iodine, such as taking iodized oil pills. So, two soil samples (5 ~ 20cm) and two peripheral water samples were randomly selected from each survey site. Each district selected 20 permanent residents over 18 years old (non-same household, male and female half), 20 pregnant women, 40 children (boy and girl half). Urine sample (10mL), salt sample (50g), thyroid B-ultrasound detection in adults and pregnant women, adult blood sample (5mL), urine iodine, salt iodine, thyroid volume and nodule detection, and thyroid function detection in adults were collected. Including free triiodothyronine (FT3), free thyroxine (FT4), thyroid stimulating hormone (TSH), thyroglobulin antibody (TGAb), thyroid peroxidase antibody (TPOAb) determination.

### 2.3 Investigation of iodine content in food

Grains (rice, noodles), meat (pork, beef, mutton, chicken, duck), milk (milk, yogurt), eggs (eggs, duck eggs, salted duck eggs), fish (freshwater fish, shellfish, sea fish, shrimp, crabs), bean products (tofu, bean curd, dried beans), seaweed (kelp, laver, nori), pickled products (Chinese sauerkraut, tuber mustard, etc.), processed food (sausage, ham sausage) 9 categories, each category 2 different kinds of samples sold in the market are collected in each county (district). Collect 5 servings of different kinds of fresh vegetables and 5 servings of different kinds of fresh fruits that are commonly eaten by local residents. No less than samples of various types of food were collected from each city, county (district) to determine the iodine content.

### 2.4 Survey of total iodine intake in population

Per capita daily salt intake, per capita daily water intake and Iodine-specific food Frequency Questionnaire (I-FFQ) were measured using 3-day weighing method to calculate the daily food intake of adults and pregnant women.

According to the following formula: Total dietary iodine intake =[(∑ intake of various types of food × iodine content of various types of food) + (drinking water + water consumption for cooking food) × iodine content of water + salt intake × salt iodine content]× (1- cooking loss rate) ×92% bioavailability. The WHO defines the cooking loss rate of iodized salt as 20%.

### 2.5 Detection method

The salt iodine content was detected by direct titration method of “Determination of iodine by General Test Method for Salt Industry”. The urinary and food iodine content was determined using the As-Ce catalytic spectrophotometry method. Determination of soil iodine content according to “Regional geochemical Sample analysis method Part 24 Inductively coupled Plasma mass spectrometry”. Thyroid function test was determined using the direct chemiluminescence method. Thyroid measurement was performed using B-ultrasound with a probe frequency of 7.5 MHz or higher.

### 2.6 Diagnostic criteria

Participants were randomly selected. The diagnostic criteria for thyroid diseases were listed in **Table 1**.

**Table 1:** Diagnostic criteria for the thyroid diseases included in this study.

Thyroid disease	Diagnostic criteria*
Goiter	Thyroid volume >25 ml (male) or >18 ml (female) (14)
Nodule	Goiter with nodule >5 mm in diameter
Overt hyperthyroidsim	FT4 > 19.05 pmol/L, TSH < 0.35 μIU/mL
Subclinical hyperthyroidsim	FT4 within the normal range, TSH < 0.35 μIU/mL
Overt hypothyroidism	TSH > 4.94 μIU/mL, FT4 < 9.01 pmol/L
Subclinical hypothyroidism	TSH > 4.94 μIU/mL, FT4 within the normal range
Thyroid autoimmunity	TPOAb > 5.61 IU/mL; TGAb > 4.11 IU/mL, or both

*Reference ranges: FT3, 2.43–6.01 pmol/L; FT4, 9.01–19.05 pmol/L; TSH, 0.35–4.94 μIU/mL; TPOAb, 0–5.61 IU/ml; TgAb, 0–4.11 IU/ml. Reference ranges were provided by the fourth affiliated hospital of Harbin Medical University.

### 2.7 Statistical analysis

Excel software was used to establish the database and organize the data. Statistical analyzes of this study were performed using IBM SPSS v20.0 (IBM Corporation, USA). Normal distribution test was carried out for continuous data, normal distribution data was represented by mean, and T-test was used for comparison between groups. Non-normal data were represented by median. Mann-whitney test was used for comparison between two groups, and Kruskal-Wallis test was used for comparison between multiple groups. Statistical data were expressed as numbers and percentages, and χ^2^ test was used for comparison between groups. Bilateral test was used in all cases, and P < 0.05 was considered statistically significant.

## 3 Results

### 3.1 Basic information

According to the geographical location of Hainan Province, the 21 districts and counties are divided into western coastal areas (Chengmai, Changjiang, Ledong, Danzhou, Dongfang, Lingao), central mountainous areas (Baoting, Tunchang, Baisha, Qiongzhong, Wuzhishan, Ding ‘an) and eastern coastal areas (Xiuying District, Longhua District, Qiongshan District, Meilan District, Qionghai, Sanya, Wanning, Wenchang, Lingshui). The median water iodine concentration in the western coastal, central mountainous, and eastern coastal areas was below 10.0 μg/L. Additionally, the coverage of iodized salt and the rate of consumption of qualified iodized salt were both over 95%. Basic information is shown in **Table 2**.

**Table 2:** Basic information of the survey areas.

Survey areas	Survey object	Water Iodine (μg/L)	Soil Iodine (mg/kg)	Urinary Iodine (μg/L)	Household salt iodine content (mg/kg)	Iodized salt coverage(%)	Qualified iodized salt consumption rate(%)
West coast	children	6.1 (672)	2.82 (60)	179.1(1238)	24.9 (1868)	98.2	96.3
	adults			141.6(603)			
	pregnant women			139.4(610)			
Central area	children	3.8 (634)	5.13 (60)	186.0(1236)	25.0 (1840)	99.2	98.0
	adults			148.4(602)			
	pregnant women			141.4(604)			
East coast	children	6.0 (1260)	5.17 (90)	172.8(1827)	24.1 (2733)	98.3	95.6
	adults			155.4(913)			
	pregnant women			132.3(906)			

(): number

### 3.2 Iodine content of food in different regions

A total of 632 food samples were collected, among which the iodine content of laver was the highest (4424.76 µg/100g), and that of condiment was the lowest (2.41 µg/100g). The difference analysis of iodine content of various foods between the western coastal area, the central mountainous area and the eastern coastal area showed that there was no statistical difference in iodine content of other foods except for the significant difference in fruit (*H*=6.656, *P*=0.036). The results are showed in **Table 3**.

**Table 3:** Iodine content of food in different regions(µg/100g).

Food	Western		Central		Eastern		Total		*H*	*P*
	N	IC	N	IC	N	IC	N	IC		
Staple food	11	4.62	12	2.89	14	3.82	37	3.76	2.948	0.229
Bean products	15	13.11	13	10.39	23	7.56	51	9.91	4.315	0.116
Meat	24	6.75	27	6.76	24	5.60	75	6.44	3.112	0.211
Eggs	15	25.87	14	26.44	14	23.62	43	25.32	0.619	0.734
Dairy	12	24.23	11	25.21	17	23.03	40	23.99	0.148	0.929
Seafood	17	32.43	20	32.15	29	23.96	66	28.16	2.270	0.321
Vegetables	33	3.31	27	4.99	45	4.49	105	3.86	3.040	0.219
Fruits	31	2.50	28	3.06	44	4.10	103	3.34	6.656	0.036
Nori	6	4386.7	6	4880.0	9	4688.9	21	4424.8	1.212	0.545
Wet kelp	6	1606.0	6	2785.0	5	2570.2	17	2305.7	0.500	0.779
Salted products	14	8.11	9	8.41	20	7.22	43	7.76	2.439	0.295
Processed food	9	45.29	6	49.48	13	47.88	28	46.42	0.115	0.944
Condiment	2	2.41	2	2.41	1	2.41	5	2.41	1.800	0.407

IC, iodine concentration.

### 3.3 Dietary iodine intake of different populations in different regions

A total of 4227 people were investigated, including 2120 adults and 2107 pregnant women. The food intakes of adults and pregnant women in western coastal areas, central mountainous areas and eastern coastal areas were 117.0 µg/d, 102.8 µg/d and 106.2 µg/d, respectively. Except soybean products, fruits and processed foods, iodine intake of other foods had statistical significance among different regions (*P*<0.05). The results are listed in **Table 4**.

**Table 4:** Dietary iodine intake of different populations in different regions (µg/d).

Food	Western		Central		Eastern		Total		*H*	*P*
	A	PW	A	PW	A	PW	A	PW		
Staple food	11.1	9.0	13.8	11.6	10.5	9.6	11.8	10.1	47.034	<0.001
Bean products	10.0	1.5	1.0	1.2	0.9	1.1	4.0	1.3	52.687	<0.001
Meat	2.0	1.9	2.3	2.3	2.2	2.2	2.2	2.1	6.322	0.012
Eggs	5.5	7.1	5.7	7.3	6.6	7.5	5.9	7.3	82.861	<0.001
Dairy	13.7	28.2	10.4	28.7	15.5	28.2	13.2	28.4	497.320	<0.001
Seafood	7.8	8.7	5.0	5.3	4.8	8.9	5.9	7.6	7.780	0.005
Vegetables	10.0	9.6	10.8	11.2	11.0	10.8	10.6	10.5	1.594	0.207
Fruits	4.1	5.9	4.0	5.9	4.8	5.5	4.3	5.8	209.566	<0.001
Nori	24.1	21.8	16.3	15.3	16.2	18.5	18.9	18.5	4.345	0.037
Wet kelp	24.8	24.3	20.8	24.4	19.2	26.6	21.6	25.1	6.823	0.009
Salted products	0.4	0.3	0.4	0.3	0.2	0.2	0.3	0.3	35.482	<0.001
Processed food	0.6	0.5	0.5	0.4	0.3	0.3	0.5	0.4	8.895	0.003
Condiment	0.1	0.1	0.1	0.1	0.1	0.1	0.1	0.1	14.730	<0.001
Total	114.2	118.9	91.1	114.0	92.3	119.5	99.2	117.5	152.015	<0.001
Utilization rate	84.1	87.5	67.0	83.9	67.9	88.0	73.0	86.5	152.024	<0.001

A, adults; PW, pregnant women; Utilization rate= Total× (1- 20%cooking loss rate) ×92% bioavailability.

### 3.4 Iodine intake in water of different populations in different regions

The median iodine concentration in total water was 6.0 μg/L, and the iodine intake through drinking water was 6.5 μg/d. The daily iodine intake in western coastal area, central mountainous area and eastern coastal area was 6.1 μg, 3.6 μg and 7.6 μg, respectively. There were statistically significant differences in water iodine intake between adults and pregnant women in different regions (*P*<0.05). The results are shown in **Table 5**.

**Table 5:** Iodine intake in water of different populations in different regions (μg/d).

Area	WI	Adults			PW			Total			H	p
		N	WII	TII	N	WII	TII	N	WII	TII		
Western	6.5	501	1001	6.5	502	910	5.9	1003	931	6.1	11.736	0.001
Central	3.3	701	1221	4.0	704	1042	3.4	1405	1083	3.6	8.863	0.003
Eastern	6.5	918	1187	7.7	901	1180	7.7	1819	1175	7.6	0.186	0.666
Total	6.0	2120	1153	6.9	2107	1056	6.3	4227	1087	6.5	14.046	<0.001

WI, water iodine; WII, water iodine intake; TII, Total iodine intake.

### 3.5 Iodine intake in salt of different populations in different regions

A total of 2120 adults and 2107 pregnant women were investigated. The median salt iodine content was 24.6 mg/kg, and there was no statistical difference in salt iodine content between different regions. The total salt iodine intake was 172.2 μg/d, and the daily salt iodine intake was 173.2 μg, 172.2 μg and 169.4 μg in western coastal area, central mountainous area and eastern coastal area, respectively, with no significant difference. The results are listed in **Table 6**.

**Table 6:** Iodine intake in salt of different populations in different regions (μg/d).

Area	SI	Adults			Pregnant women			Total			H	p
		N	SII	TII	N	SII	TII	N	SII	TII		
Western	24.4	601	6.9	168.4	602	6.9	168.4	1203	7.1	173.2	0.006	0.938
Central	24.6	601	7.0	172.2	604	6.9	169.7	1205	7.0	172.2	1.316	0.251
Eastern	24.2	918	7.1	171.8	901	7.3	176.7	1819	7.0	169.4	1.766	0.184
Total	24.6	2120	7.0	172.2	2107	7.0	172.2	4227	7.0	172.2	2.061	0.151

SI, salt iodine; SII, Salt iodine intake; TII, Total iodine intake.

### 3.6 Total iodine intake and distribution of different populations in different regions

Excluding 20% cooking loss rate and considering 92% bioavailability, the average total iodine intake of residents was 211.5 μg/d, that of adults 204.8 μg/d, and that of pregnant women 217.9 μg/d. The total daily iodine intakes were 218.1 μg, 205.0 μg and 208.4 μg in the western coastal region, the central mountainous region and the eastern coastal region, respectively. There were statistically significant differences in total iodine intake among different populations in different regions (*P*<0.05). The results are shown in **Table 7**.

**Table 7:** Total iodine intake and distribution of different populations in different regions (μg/d).

Area	Western			Central			Eastern			Total		
	A	PW	Total	A	PW	Total	A	PW	Total	A	PW	Total
Food	114.2	118.9	117	91.1	114	102.8	92.3	119.5	106.2	99.2	117.5	108.7
Water	6.5	5.9	6.1	4	3.4	3.6	7.7	7.7	7.6	6.9	6.3	6.5
Salt	168.4	168.4	173.2	172.2	169.7	172.2	171.8	176.7	169.4	172.2	172.2	172.2
Total	289.1	293.2	296.3	267.3	287.1	278.6	271.8	303.9	283.2	278.3	296.0	287.4
Utilization rate	212.8	215.8	218.1	196.7	211.3	205.0	200.0	223.7	208.4	204.8	217.9	211.5

A, adults; PW, pregnant women; Utilization rate= Total× (1- 20%cooking loss rate) ×92% bioavailability.

### 3.7 Urinary iodine status of different populations in different regions

The overall median urinary iodine levels in children, pregnant women and adults were 180.3 μg/L, 144.6 μg/L and 151.6 μg/L, respectively, and the difference was statistically significant (*P*<0.05). The median urinary iodine values of children in the central mountainous area were 190.8 μg/L, 149.4 μg/L and 161.0 μg/L, respectively, which were significantly higher than those in the western coastal area and the eastern coastal area. The results are shown in **Table 8**.

**Table 8:** Median urinary iodine status of different populations in different regions.

Area	Children	Pregnant women	Adults	Z	P
Western	184.2	137.0	144.9	117.92	<0.05
Central	190.8	149.4	161.0	121.42	<0.05
Eastern	174.3	145.6	149.8	80.051	<0.05
Total	180.3	144.6	151.6	314.8	<0.05
Z	14.364	7.633	11.354		
P	0.001	0.022	0.003		

### 3.8 Prevalence of thyroid diseases in adults in different region

A total of 2078 adults were surveyed, including 940 males and 1138 females. The detection rates of different thyroid diseases were: thyroid nodule (25.41%), subclinical hyperthyroidism (3.66%), subclinical hypothyroidism (2.60%), goiter (2.45%), over hypothyroidism (1.36%) and over hyperthyroidism (0.77%). The positive rates of TgAb and TPOAb were 26.95% and 20.40%, respectively. There were significant differences in thyroid nodules in the western coastal area, the central mountainous area and the eastern coastal area. No statistical difference was found in different regions for other diseases. The results are shown in **Table 9**.

**Table 9:** Prevalence of thyroid diseases in adults in different region (%).

Diseases	Western (n=897)		Central (n=586)		Eastern (n=897)		TDR	*χ^2^*	*p*
	N	DR	N	DR	N	DR			
over hypothyroidism	2	0.34	4	0.68	10	1.11	0.77	2.699	0.237*
subclinical hypothyroidism	14	2.35	13	2.22	27	3.01	2.6	1.076	0.584
over hyperthyroidism	6	1.01	3	0.51	12	1.34	1.01	2.417	0.299
subclinical hyperthyroidism	22	3.7	24	4.1	30	3.34	3.66	0.571	0.752
thyroid nodules	139	23.36	197	33.62	192	21.4	25.41	29.739	<0.001
goiter	10	1.68	12	2.05	29	3.23	2.45	4.164	0.125
positive TPOAb	122	20.5	111	18.94	191	21.29	20.40	1.212	0.546
positive TgAb	171	28.74	157	26.79	232	25.86	26.95	1.513	0.469

DR, detection rates.

## 4 Discussion

The average iodine content in soil and the median iodine content in water were 4.37 mg/kg and 6.0 μg/L respectively. According to the Standard for the Definition and demarcation of iodine deficient areas and iodine adequate areas (WS/T 669-2020) (7) all cities, counties and towns in Hainan Province are iodine-deficient areas. The iodine content in soil and water was low, and there was no correlation between the two.

According to the results, the coverage rate of iodized salt was 98.6%, and the consumption rate of qualified iodized salt was 97.9%. The iodine content of various foods in Hainan Province is similar to the results of the Iodine Supplement Guide for Chinese Residents, and the average dietary iodine intake of residents is 211.5 μg/d (Adults 204.8 μg/d, pregnant women 217.9 μg/d), slightly lower than the 231.22 μg/d in coastal areas of Tianjin (8) and slightly higher than the 198.3 μg/d in coastal areas of Fujian (9). According to the Reference Intake of Dietary Nutrients for Chinese Residents (2023 edition), the dietary iodine intake of the general population of Hainan residents is within the UL (Tolerable upper intake level) (600 μg/d), while that of pregnant women is slightly lower than RNI (Recommended nutrient intake) (230 μg/d).

The iodine intake of Hainan residents mainly comes from salt iodine, and the contribution rate of salt iodine to total dietary iodine is 59.9% (172.2/287.4), which is lower than that of Tianjin (8) (69.0%) and Fujian (9) (63.8%). The food with high iodine intake is kelp, whose contribution rate is 8.1% (23.4/287.4), and the total contribution rate of other foods is 32%. Among them, the contribution rate of fish, shrimp and crabs and other seafood is only 2.4%, ranking seventh among the dietary iodine contribution rates of Hainan residents. The contribution rates of processed food, pickled products and condiments were only 0.2%, 0.1% and 0 respectively, mainly because the processed food and pickled products of Hainan residents generally used non-iodized salt, and soy sauce was the main condiment.

Hainan Province is located in the coastal zone, seafood sources are rich, and the overall seafood intake of residents is higher, but the contribution rate of aquatic products to the dietary iodine of Hainan residents is far lower than that of iodized salt, kelp, seaweed. It can be seen that in coastal areas, although the iodine intake in some foods is higher than that in inland areas, the iodine nutrition obtained from food alone still does not reach the recommended intake, This is consistent with other research findings (10). Compared with the reference intake of dietary nutrients (DRIs), if there is no salt iodine intake, 90.1% of Hainan residents’ dietary iodine intake is less than RNI, and 9.9% is between RNI and UL. When salt iodine intake was added, the dietary iodine intake was less than the RNI in 34.7% of the individuals. If no iodized salt was consumed, the dietary iodine intake of the vast majority of adults (85.0%) and pregnant women (95.1%) could not reach the RNI. After adding iodized salt, 97.7% of the adult dietary iodine intake in Hainan Province was between RNI and UL, and 67.5% of the pregnant women’s dietary iodine intake was lower than the RNI formulated by the Chinese Nutrition Society. Due to the special physiological needs of pregnant women, RNI is higher than the general population, under the same salt iodine level, cannot meet the needs of the body, pregnant women should be guided to increase the intake of iodine-rich food.

As a key group of iodine supplementation, it is of great significance for children to maintain appropriate iodine nutrition status, and the median urinary iodine of children is often used as an indicator of iodine nutrition evaluation of the whole population. The survey shows that the median urinary iodine of children in Hainan Province is 180.3 μg/L, which is at an appropriate level, the ratio of urinary iodine < 50 μg/L is 4.3%, and the median urinary iodine of adults is 151.6 μg/L, which meets the elimination standard of IDD in China, indicating that through the cooperation of relevant departments and joint efforts in various aspects, with the intervention of comprehensive prevention and control measures based on USI (Universal salt iodization), the iodine nutrition level of Hainan residents remained in a suitable state. It also confirmed the rationality of using the urinary iodine level of children as the iodine nutrition evaluation index of the general population in the national iodine deficiency elimination monitoring system.

The monitoring results showed that the median urinary iodine of pregnant women in the province was 144.6 μg/L, slightly lower than the recommended standard, and there was a risk of iodine deficiency, which should be paid great attention to. The median urinary iodine was lower than the Kerver JM’s result (11). The median urine iodine in 6 of the 21 districts or counties was greater than 150.0 μg/L, and the median urine iodine in the other 15 cities or counties was between 100.0 and 149.0 μg/L. Pregnant women, as a special needs population, may need other iodine supplementation measures to meet their own needs and those of their offspring. Some studies have also shown that the median urinary iodine between 100 and 149 µg/L can meet the iodine needs of pregnant women, and the difference in urine volume during different pregnancy may affect the median urinary iodine. Given the high iodized salt coverage rate, scientific research on the appropriate median urinary iodine should be further strengthened.

The overall prevalence rate of adult thyroid disease in this survey was 27.01% (550/2036), which was similar to that of the study conducted by Weiping Teng (12, 13). The prevalence of subclinical hypothyroidism and subclinical hyperthyroidism in males was significantly higher than that in females, while the detection rate of thyroid nodules and goiter was significantly lower than that in females, this is similar to Liu’s findings (14). The detection rate of thyroid nodules was significantly different among different regions, and the central mountainous area was significantly higher than the eastern and western coastal areas. There was no regular trend in the detection rates of other diseases in the three regions, and no statistical difference was observed in all the three regions, which could not be attributed to the fact that urinary iodine in the central mountainous region was significantly higher than that in the other two regions (161.0 μg/L VS149.8 μg/L, 144.9 μg/L), and the median urinary iodine in the three regions was within the appropriate range. The pathogenesis of thyroid diseases is complicated and varied, and may be affected by heredity, endocrine dysfunction, immune dysfunction, mental stress, environmental pollution and other factors (15–18). Currently, the detection rate of thyroid diseases in Hainan Province is at a normal level.

However, this research still has some limitations. For example, the small size of the analyzed population groups, the heterogeneity of the evaluated samples, the variability of foods in the diet of the locals included in the study, etc.

In conclusion, the level of iodine in ambient water and soil is low in all districts or counties of Hainan Province, all of which are iodine deficient areas, and the iodine intake of residents mainly comes from salt. The iodine nutrition level of children and adults is in an appropriate state, and the median urine iodine of pregnant women is slightly lower than the recommended standard. Pregnant women can appropriately add iodine-rich foods such as kelp and laver when eating iodized salt. The overall detection rate of thyroid disease was in a reasonable range, and no statistical difference was found between different regions except for the detection rate of thyroid nodules. Therefore, salt iodization should be insisted on preventing IDD in all island regions of China.

## Data Availability

The original contributions presented in the study are included in the article/supplementary material. Further inquiries can be directed to the corresponding authors.
